# Long COVID and its association with neurodegenerative diseases: pathogenesis, neuroimaging, and treatment

**DOI:** 10.3389/fneur.2024.1367974

**Published:** 2024-04-04

**Authors:** Jinyang Zhao, Fan Xia, Xue Jiao, Xiaohong Lyu

**Affiliations:** ^1^Department of Radiology, The First Affiliated Hospital of Jinzhou Medical University, Jinzhou, China; ^2^Department of Respiratory, The First Affiliated Hospital of Jinzhou Medical University, Jinzhou, China

**Keywords:** long COVID, SARS-CoV-2, neurodegenerative diseases, Parkinson’s disease, neuroimaging

## Abstract

Corona Virus disease 2019 (COVID-19), caused by the severe acute respiratory syndrome coronavirus-2 (SARS-CoV-2), has presented unprecedented challenges to the world. Changes after acute COVID-19 have had a significant impact on patients with neurodegenerative diseases. This study aims to explore the mechanism of neurodegenerative diseases by examining the main pathways of central nervous system infection of SARS-CoV-2. Research has indicated that chronic inflammation and abnormal immune response are the primary factors leading to neuronal damage and long-term consequences of COVID-19. In some COVID-19 patients, the concurrent inflammatory response leads to increased release of pro-inflammatory cytokines, which may significantly impact the prognosis. Molecular imaging can accurately assess the severity of neurodegenerative diseases in patients with COVID-19 after the acute phase. Furthermore, the use of FDG-PET is advocated to quantify the relationship between neuroinflammation and psychiatric and cognitive symptoms in patients who have recovered from COVID-19. Future development should focus on aggressive post-infection control of inflammation and the development of targeted therapies that target ACE2 receptors, ERK1/2, and Ca^2+^.

## 1 Long COVID

COVID-19 is a multi-system disease caused by infection with SARS-CoV-2 virus (1). As the virus mutates, its virulence and transmissibility gradually decrease, resulting in reduced mortality and risk of severe and critical illness to varying degrees (2). However, it is important that a significant number of individuals do not fully recover from the disease.

According to recent reports, more than two-thirds of COVID-19 patients who have been hospitalized do not fully recover even after several months of hospitalization (3). A significant number of individuals who have recovered from acute COVID-19 experience a sustained immune response and chronic inflammation, which can lead to severe tissue damage (4). Unlike acute COVID, there are currently no consistent guidelines for managing long COVID. The World Health Organization’s Delphi Consensus defines long COVID as enduring symptoms in individuals who have previously been infected with SARS-CoV-2 and experience symptoms that last for at least 2 months, with no explanation from an alternate diagnosis. These symptoms can occur either during the initial recovery phase or persist after the initial infection. They may also fluctuate or recur over time (5). The most common systemic symptoms of long COVID include fatigue, limb weakness, generalized pain, neurological and cognitive-psychological issues, and cardiopulmonary dysfunction (6–13).

The nervous system plays a crucial role in the long-term effects of COVID-19. Studies have shown that a significant percentage (30–80%) of COVID-19 patients have experienced neurological sequelae or changes in mental health (14). A large study conducted in the United States found that COVID-19 survivors with cerebrovascular or neurodegenerative diseases, such as stroke, Alzheimer’s disease, or Parkinson’s disease, were about 40% more likely to experience these sequelae compared to individuals without SARS-CoV-2 infection (15). Cognitive disturbances and fatigue are particularly prominent and debilitating symptoms of long COVID, which are also significant indicators in neurodegenerative diseases.

## 2 Possible mechanisms of long-term effects on the nervous system

**Figure 1:**
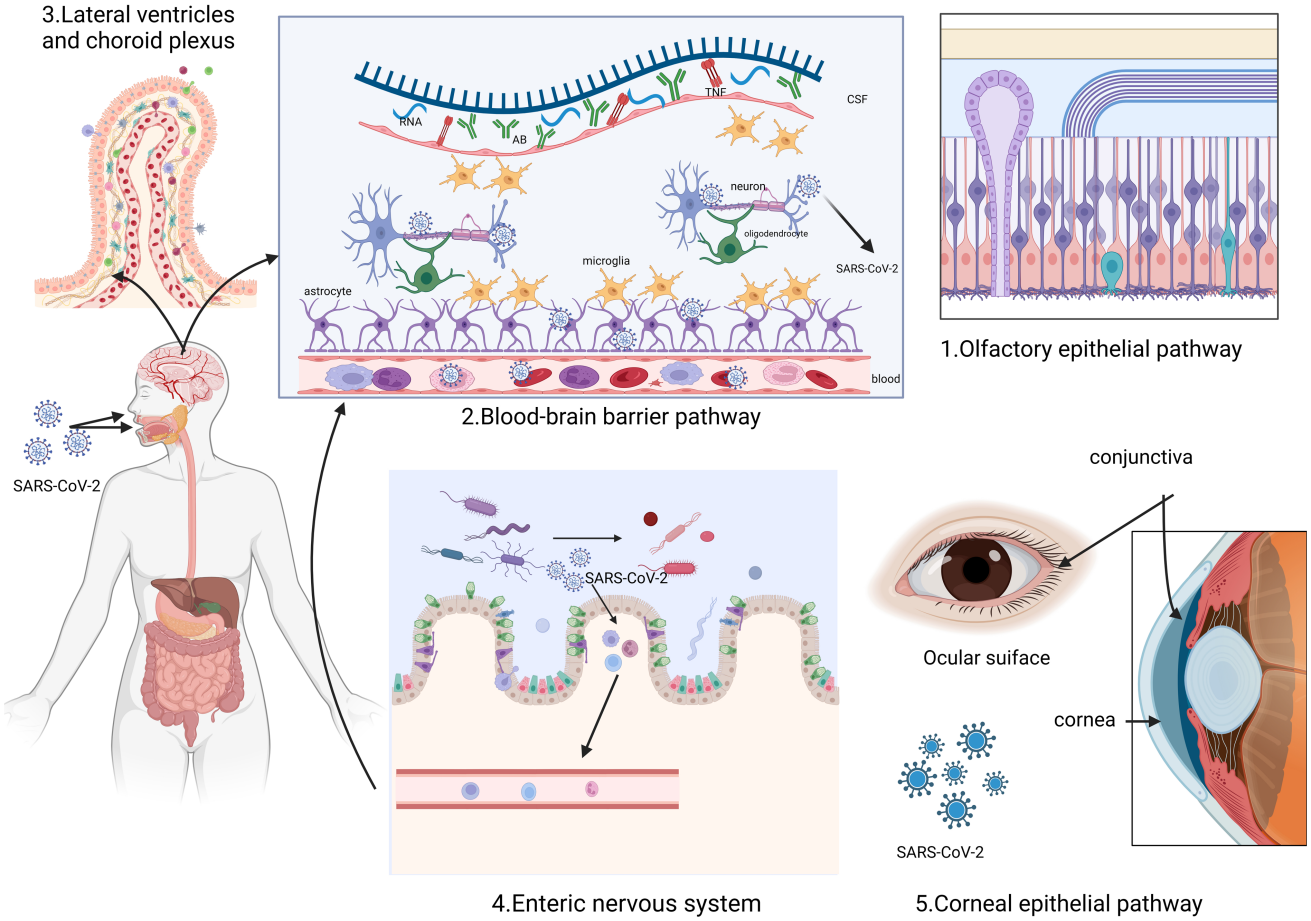
This figure illustrates the five routes through which SARS-CoV-2 enters the nervous system. It can enter through the olfactory epithelium and spread through the olfactory nerve (1). Additionally, it can pass through the blood–brain barrier (2) and the lateral ventricular choroid plexus (3) due to the interaction of inflammatory factors. Another possible route is through the microorganism gastrointestinal tract-brain axis (4). Furthermore, the corneal epithelium of the optic nervous system can also act as an entry point (5). Created with BioRender.com.

The SARS-CoV-2 enters human cells by interacting with specific membrane cell receptors, such as angiotensin-converting enzyme 2 (ACE2) transmembrane receptor, and activating SARS-CoV-2 spike protein through transmembrane serine protease 2 (TMPRSS2) cleavage (16, 17). Evidence suggests the presence of the virus in the nervous system, with the first case of SARS-CoV-2-associated meningitis reported by Moriguchi (18). Additionally, SARS-CoV-2 has been detected in cerebrospinal fluid by other researchers (19, 20). This section summary outlines the potential routes of entry into the nervous system by SARS-CoV-2 and the possible mechanisms underlying long COVID (Figure 1).

### 2.1 Olfactory epithelial pathway

In the majority of COVID-19 cases, a considerable number of patients initially experience a reduced sense of smell (hyposmia) or complete loss of smell (anosmia). This can be attributed to damage to the olfactory epithelium caused by the SARS-CoV-2 virus. The damage then affects the olfactory neural network, which is connected to the primary olfactory cortex (3, 5, 21). The study suggests that SARS-CoV-2 may pass through nerves within the olfactory mucosa, potentially creating a pathway for nerve invasion through the mucosal interface into the nervous system (21, 22).

The inflammatory pathways triggered by SARS-CoV-2 in the nasal epithelium show significant similarities with the inflammatory signaling observed in certain groups of patients with dementia (23). First, olfactory impairment is probably one of the most common early clinical manifestations of neurodegenerative diseases and COVID-19. The olfactory mucosa can act as a pathway for SARS-CoV-2 to invade the central nervous system (CNS) through axonal transport (23). Furthermore, ACE2, a cell surface receptor responsible for S protein-mediated entry of SARS-CoV-2, is expressed by epithelial cells in the human olfactory mucosa (24). The extent of α-synuclein (α-syn) lesions in other brain regions is strongly correlated with the pathological burden of the olfactory bulb, indicating that the lesions of Parkinson’s disease (PD) extend along the olfactory pathway (25). According to the Braak hypothesis, Lewy bodies (LB) initially appear in olfactory structures, such as the olfactory bulb, and then gradually spread to the brainstem and eventually the cerebral cortex. This supports the possibility that the earliest lesions may develop in regions other than the substantia nigra (26, 27). Consequently, an inflammatory stimulus originating from the nasal epithelium and affecting the olfactory bulbs and interconnected brain regions could potentially expedite the progression of neurodegenerative diseases and their associated pathological processes.

### 2.2 Blood–brain barrier pathway

The blood–brain barrier (BBB) can serve as a potential route for SARS-CoV-2 to enter the brain. This can occur through two pathways: the vascular endothelial cell pathway and the immune cell pathway. The permeability of the BBB is regulated by tight junctions in vascular endothelial cells. The presence of ACE2 in systemic vascular endothelial cells provides a molecular basis for how SARS-CoV-2 can breach the BBB and infect the brain (28). SARS-CoV-2 attaches to ACE2 receptors (29), and enters vascular endothelial cells through endocytosis and exocytosis, allowing for cell-to-cell transmission of the virus (28). Studies have shown that SARS-CoV-2 can interact with macrophages, microglia, and astrocytes in the CNS, triggering the release of cytokines and causing high inflammation (30). This inflammatory state can result in increased permeability of the BBB, dysfunction of cerebrovascular endothelial cells, and disruption of BBB integrity. Consequently, more inflammatory factors can enter the CNS, impacting cognitive function and potentially leading to the development of neurodegenerative diseases (31). These effects are supported by histopathological examinations of the brains of SARS-CoV-2-infected patients, which revealed the presence of CD^3+^ T lymphocytes and CD^68+^ monocytes/macrophages within the brain’s mesenchymal cells (32).

### 2.3 Lateral ventricles and choroid plexus

According to McQuoid and colleagues (29), the lateral ventricles and choroid plexus could potentially be entry points for SARS-CoV-2 into the CNS. They propose that ACE2-expressing epithelial cells in these areas may aid in the passage of the virus across the blood-cerebrospinal fluid barrier and into the choroid plexus and ventricular system. This provides important histological evidence supporting the neuroinvasive nature of SARS-CoV-2 (33). However, autopsy findings have shown no presence of SARS-CoV-2 RNA and protein in brain tissue (33, 34).

### 2.4 Vagus nerve of the gastrointestinal

The enteric nervous system has been identified as the primary region where abnormal α-syn aggregation occurs, which can potentially spread from the periphery to the central nervous system (35, 36). Specifically, the dorsal motor nucleus of the vagus nerve (DMV) receives signals from vagus parasympathetic neurons and projects them throughout the gastrointestinal system. DMV is involved in the PD-neuroanatomical pathway, and in postmortem PD studies, DMV and the vagus nerve itself are commonly affected structures (37, 38). They are also the main areas where LB accumulation occurs, even in the early stages of disease development. *In vitro* studies have shown that pathological α-syn can spread from the gut to the brain via the vagus nerve, with DMV being the first affected region in the brain (39). From there, α-syn can spread to other PD brain regions, including Substantia nigra pars compacta, leading to the loss of dopaminergic neurons and the emergence of a Parkinson’s disease phenotype (40). Additionally, SARS-CoV-2 has been found in neuronal cells of the intestinal muscle plexus (41). The vagus nerve is also believed to provide a pathway for retrograde invasion of the central nervous system by SARS-CoV-2, potentially enhancing its neuro-aggressiveness (42, 43).

### 2.5 Corneal epithelial pathway

Recent studies have shown that ACE2 expression is relatively high in the corneal epithelium, indicating that COVID-19 can potentially be transmitted through the ocular or conjunctival route (44, 45). Several sampling studies have detected SARS-CoV-2 RNA in different parts of the visual system, such as the retina, optic nerve, conjunctiva, and vitreous, in patients diagnosed with SARS-CoV-2 (46, 47). These findings provide insights into the possible pathways of SARS-CoV-2 entry into the nervous system.

### 2.6 The link between long COVID and the nervous system

The mortality rate of long COVID may be associated with the disease’s severity, and it is worth noting that even a small number of individuals with mild COVID-19 can develop long COVID. A study conducted over the course of 1 year followed mild patients and found that one-third of them continued to experience symptoms of long COVID (48). Polymorphisms in ACE2 and TMPRSS2 may contribute to long COVID. Studies have shown that alterations in polymorphisms within ACE2-spike protein interactions, the proteolytic cleavage site TMPRSS2, and ACE2 expression are correlated with the susceptibility and severity of COVID-19. Certain ACE2 variants have been found to have a 28-fold increase in severe disease (49, 50). Additionally, in patients previously hospitalized with COVID-19, ACE2 susceptibility and TMPRSS2 polymorphisms were found to be associated with disease severity and the occurrence of long COVID symptoms (51). In the Post-Discharge COVID-19 Study (52) and other cohort studies, a significant association has been found between systemic inflammation and cognitive impairment in Neurological Long COVID (Neuro-LC) patients. Although the virus does not persist in neurons, it can easily infect and activate astrocytes and microglia (53). Activation of these glial cells is a characteristic of neuroinflammation, which can lead to localized brain atrophy. These findings, reported in the case of Neuro-LC, are associated with cognitive deficits (21, 54). Several studies have also identified the presence of inflammatory mediators, such as TNF, in the cerebrospinal fluid of Parkinson’s disease patients and the brains of autopsy patients. TNF, IL-6, IL-1β, and IFN-γ have been detected in these patients as well (55). As a result of direct or indirect antagonism of IL-6 via the JAK-STAT pathway, it has been shown to improve the prognosis of hospitalized COVID-19 patients with hypoxia and systemic inflammation, IL-6 may be a biomarker closely related to treatment (56). However, there is currently insufficient evidence that SARS-CoV-2 replicates within the central nervous system. Further researches are needed to gather more conclusive evidence.

## 3 The association between major neurodegenerative diseases and SAR-COV-2

Existing evidence indicates that COVID-19 has the potential to cause damage to the neurons, thereby potentially contributing to the onset of chronic degenerative diseases of the nervous system (57). A recent study revealed that individuals who had contracted COVID-19 had a greater likelihood of developing Alzheimer’s disease (AD), PD, and multiple sclerosis (MS) 6 months after infection, as compared to those affected by influenza or other respiratory infections (58). It is plausible that COVID-19 could exacerbate pre-existing conditions or even trigger subclinical neurodegenerative diseases.

### 3.1 Alzheimer’s disease

Individuals with AD have a higher susceptibility to contracting SARS-CoV-2 and a greater risk of mortality compared to those with non-cognitive impairment (59). This could be due to the combined effect of pre-existing neuroinflammatory markers in AD and the inflammatory response triggered by COVID-19, which worsens the condition following infection. Additionally, certain serological markers associated with AD, such as serum total tau, phosphorylation tau-181, Ubiquitin carboxy-terminal hydrolaseL1, Neurofibrillary acidic protein, and Neurofilamentlightchain, are positively correlated with increased infection severity (59). These biomarkers are elevated to levels similar to those observed in AD dementia and may indicate worse outcomes among hospitalized COVID-19 patients. Recent studies have also shown that neuroinflammation plays a crucial role in the development of AD, particularly in the context of SARS-CoV-2 infection. The abnormal immune response and resulting inflammation are believed to contribute to degenerative lesions and increase vulnerability to AD (60).

### 3.2 Parkinson’s disease

Parkinson’s disease is characterized by the gradual loss of dopaminergic neurons in the nigrostriatal body and the accumulation of α-syn containing LB and Lewy synapses. People with Parkinson’s disease may be more vulnerable to SARS-CoV-2 infection, which can worsen the advancement of the disease. A recent 15 months cohort study conducted by Zenesini et al. revealed that patients with Parkinson’s disease had a greater risk of SARS-CoV-2 infection and a higher likelihood of hospitalization for Parkinsonism (58%) compared to healthy controls (61). Moreover, the study also found a higher prevalence of COVID-19 in the PD population compared to the general population, although there was no significant difference in mortality rates between PD and non-PD patients (62). However, it is important to note that age and age-related comorbidities are important factors to consider in the PD population, as patients with PD are typically over 60 years old, and increasing age is associated with higher mortality rates in COVID-19 patients (63). Therefore, pre-existing comorbidities like hypertension, diabetes, and heart failure should be taken into account as confounding risk factors for severe COVID-19 in patients with PD (64).

Currently, there is no direct evidence linking SARS-CoV-2 to the development or acceleration of PD. However, it is important to note that ACE2 receptors, which are associated with COVID-19 infections, are widely expressed in various areas of the CNS. Including not only the heart-lung center of the medulla but also in the striatum where dopamine neurons are located, which findings suggest a correlation with PD (65). In addition, the E protein of brain-infiltrating SARS-CoV-2 has been speculated to induce Toll-like receptors (TLR2) activity in microglia, thereby increasing TLR2’s sensitivity to α-syn and Aβ oligomers. This suggests that TLR2 could be a target for SARS-CoV-2 infection, affecting both AD and PD (66).

### 3.3 Multiple sclerosis

Multiple sclerosis (MS) is an inflammatory and demyelinating disease that arises from an autoimmune disorder. MS is typically suspected when a patient presents with clinically isolated syndrome, which can manifest as either monosymptomatic or polysymptomatic depending on the location of the prominent lesion. The most frequently observed manifestations of MS include optic neuritis, brainstem, and spinal cord syndromes. However, there are also several less common manifestations, including cortical manifestations such as dominant parietal syndrome levy.

In MS, there is an increase in the expression of proinflammatory cytokines (IFNγ, TNF, IL2, and IL22) as well as molecules associated with sustained B lymphocyte activity and lymphoid neogenesis (CXCL13, CXCL10, LTα, IL6, and IL10). These factors contribute to elevated levels of inflammation observed in the meninges and cerebrospinal fluid of postmortem MS cases. Furthermore, a similar inflammatory response, characterized by increased levels of CXCL13, TNF, IFNγ, CXCL12, IL6, IL8, and IL10, has been detected in the cerebrospinal fluid of MS patients who exhibit high levels of gray matter damage at the time of diagnosis. These findings suggest that neuroinflammation may play a role in the neurodegenerative phase of MS (67). A prospective cohort study from the UK MS registry analyzed data from 599 MS patients infected with COVID-19.The study found that 29% of the patients experienced symptoms that lasted for more than 4 weeks, while 12.4% had symptoms that persisted for more than 12 weeks. The most commonly reported symptom was new or worsening fatigue. These findings suggest that individuals with high neurological impairment before contracting COVID-19 may be more susceptible to experiencing long-term effects of the virus. However, a study by Etemadifar and colleagues focused on patients with long-term relapsing–remitting multiple sclerosis and did not find any increase or worsening of clinical disease activity after COVID-19 (68). These differing results could be attributed to the variability in MS relapses. For instance, Garjani et al. defined MS exacerbations without considering the stable state of the previous 30 days (lasting at least 24 h) and the absence of fever, infection, or steroid use. Nevertheless, reports and theories suggesting that SARS-CoV-2 may contribute to the progression of MS should not be disregarded unless larger studies refute them (69). The inconsistency in findings can be explained by the rarity of neuroinvasive SARS-CoV-2 infection and the limited and observational nature of the conducted studies.

## 4 Neuroimaging and electrophysiology

Understanding the acute and long-term effects of COVID-19 on brain structure may offer valuable insights into neurodegenerative diseases. In this study, we examine evidence from the three most commonly used diagnostic methods in the current long COVID literature: magnetic resonance imaging (MRI) fluorodeoxyglucose positron emission tomography (FDG-PET), and electroencephalography (EEG).

### 4.1 MRI

A study conducted over three months found that the recovery phase of COVID-19 may cause disruptions in the brain’s microstructure and functional integrity, indicating potential long-term effects of SARS-CoV-2 (70). Notably, a comprehensive longitudinal study conducted in the UK Biobank involving 401 individuals who had undergone brain scans before the pandemic, compared to 384 uninfected controls, provided strong evidence for structural changes in the grey matter of COVID-19 patients (71). This study revealed reduced cortical thickness in brain areas functionally correlated to the primary olfactory cortex such as the left par hippocampal gyrus, bilateral orbitofrontal cortex, anterior cingulate cortex, temporal pole, insula, and supramarginal gyrus in those who had COVID-19 (72).

There is a limited number of imaging cases of patients with neurodegenerative diseases in the context of long COVID. A systematic review of 13 patients with post-COVID-19 parkinsonism found that the neuroimaging findings varied among the patients. Seven patients had unremarkable brain MRI, while one patient showed thalamic and pons T2/FLAIR hyperintensities with hemosiderin deposition. Another patient had mild cortical atrophy, and four patients had basal ganglia lesions (73). Additionally, a separate study reported six subjects who developed Parkinson’s disease after COVID-19, but their MRI scans did not show any remarkable findings (74). Therefore, it is suggested that the MRI findings of patients with COVID-19-related PD are not specific. Further research with a larger sample size is needed to gain more insights.

### 4.2 FDG-PET

Molecular imaging may provide detection of the severity of neural degenerative diseases. According to two related studies (75), long COVID patients displayed hypometabolism in various brain regions, including the bilateral orbitofrontal cortex, bilateral medial temporal lobes (including the hippocampus and amygdala), right thalamus, brainstem, and cerebellum. Another study examined changes in brain metabolism in COVID-19 patients compared to healthy controls (a total of 32 individuals) at different time points: during the acute phase, 1 month, and 6 months after the onset of COVID-19 (76).

**Figure 2:**
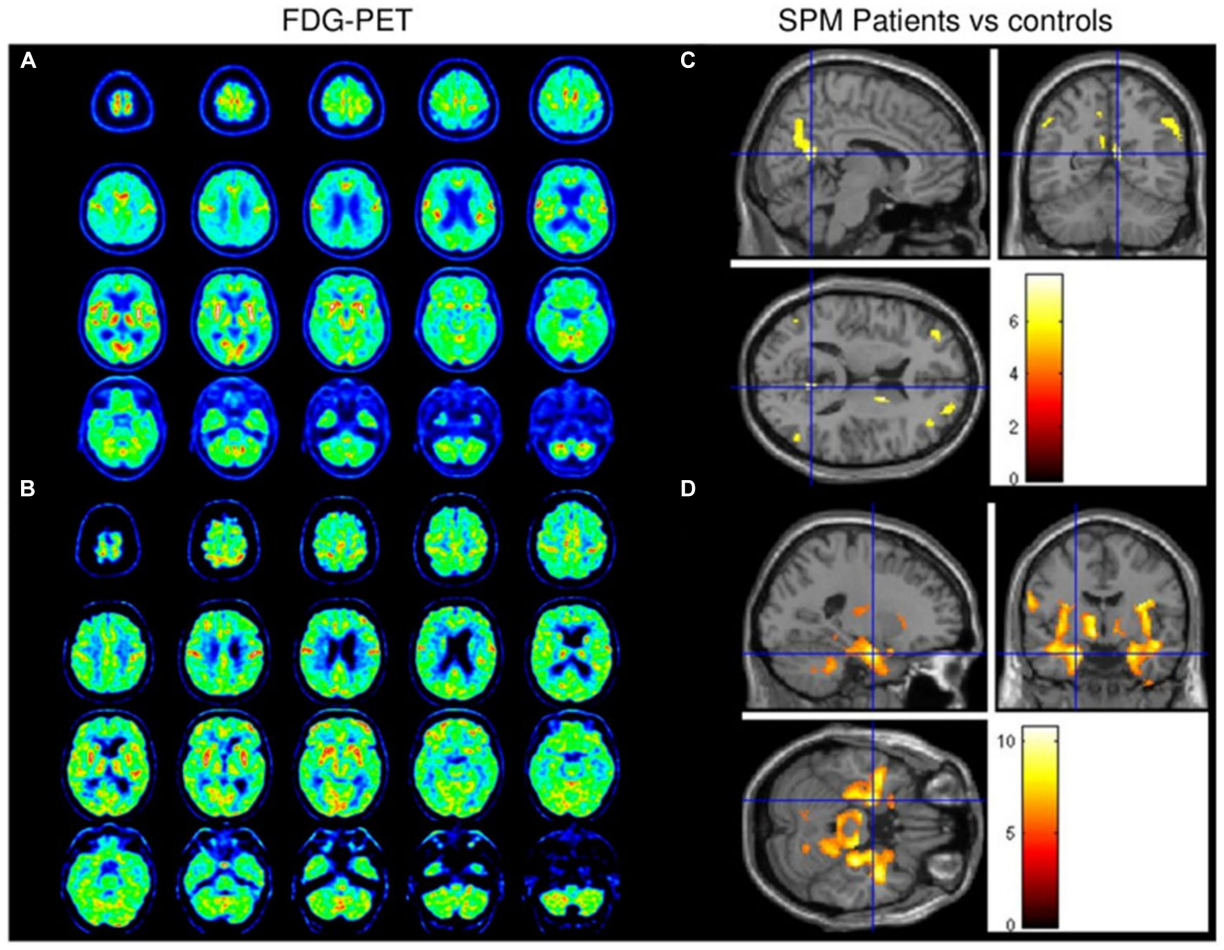
Brain FDG-PET data from patients 1 **(A)** and 2 **(B)** revealed diffuse cortical hypometabolism with relatively preserved metabolism in the sensorimotor cortex. Hypometabolism was observed in the bilateral dorsolateral prefrontal cortex, right caudate nucleus, and bilateral posterior parietal cortex **(C)**. Marked relative hypermetabolism was observed in the bilateral middle temporal cortex, basal ganglia, brainstem, and cerebellum **(D)**. Reprinted with permission, Morassi et al. SARS-CoV-2-related encephalitis with prominent parkinsonism: clinical and FDG-PET correlates in two patients (79).

One of the earliest and most common symptoms of PD and AD is impaired sense of smell. A case-control study found that patients with long COVID showed decreased metabolism in specific areas of the brain, such as the right Parhippocampal gyrus, thalamus, orbitofrontal cortex, or brainstem (substantia nigra). These changes in brain metabolism may be associated with symptoms such as loss of smell, advanced age, or fatigue (77). Therefore, COVID-19 may cause degenerative changes in the brain through the loss of sensory input, nervous system inflammation, or olfactory pathway dysfunction. In addition, a nigrostriatal dopaminergic deficit was suspected in patients 2 to 8 weeks after COVID-19 with ^123^I-DaTscan SPECT (78, 79) (Figure 2) and ^18^F-F-DOPA PET (80) with some case reports. As neurodegenerative diseases progress slowly, the short-term outcome observed in this study could be attributed to underlying preclinical Parkinson’s disease (PD) (81). The uncertainty surrounding the pre-infectious neurological condition of PD patients after contracting COVID-19 raises important questions about establishing a causal relationship between the two. Of the 13 patients included in a review, who performed dopaminergic functional imaging presented with an altered presynaptic dopaminergic tracer binding (73). At present, one ongoing Phase II/III study is attempting to quantify neuroinflammation using PET imaging in recovered COVID-19 patients in relationship to psychiatric and cognitive symptoms (82). To better understand the long-term effects of COVID-19 on the nervous system, further longitudinal studies using FDG-PET imaging should be conducted.

### 4.3 EEG

EEG is a valuable non-invasive tool for assessing neuronal activity and can be used as a functional marker for identifying synapse dysfunction and loss in cognitive impairment (83). It is important to note that even individuals with normal MRI scans may exhibit abnormal cortical activity on EEG (84, 85). After SARS-CoV-2 infection, it has been observed that many individuals show EEG abnormalities, including generalized slowing and epileptiform discharges, particularly in the frontal region (85–87). The relationship between these EEG abnormalities and cognitive dysfunction is still under investigation, with some studies finding no direct link while others have identified associations with performance on tests measuring frontal functions like the frontal assessment battery and the trail-making test (84). These findings suggest that EEG abnormalities in the frontal region could potentially serve as biomarkers (86). In a study conducted on the COVID-19 group 6–12 months after acute infection, a decrease in signal complexity was observed in the F3–F7 areas during rest. Additionally, cognitive function worsened during this period, and there were correlations between nonlinear EEG features and cognitive test results (88).

## 5 Potential treatments

Due to the lack of a unified statement on the pathogenesis and diagnostic criteria of long COVID, the development of systematic and standardized treatment methods remains challenging. Currently, there is no validated evidence-based therapy, and patient management primarily focuses on symptomatic treatment and multidisciplinary cooperation. A recent analysis of over 200 long COVID symptoms in more than 9,000 individuals has resulted in the creation of a scoring algorithm. This algorithm offers a diagnostic framework for long COVID and proposes defining it as a distinct disease specific to SARS-CoV-2 infection (89). This definition will aid in future research on the potential mechanisms, prevention, and treatment interventions in clinical and experimental studies.

### 5.1 Control of inflammation after infection

Controlling inflammation post-infection can help alleviate prolonged cytokine release, activation of immune cells, and pronounced neuroimmune responses, thereby reducing neurological symptoms associated with long COVID. Studies have shown a decreased incidence of long COVID in patients with a less strong inflammatory and immune response to acute infection, including those who have received vaccination (90) and patients who have taken antiviral medications (91, 92). While vaccination is highly effective in reducing the severity and mortality of COVID-19 and provides short-term protection against infection, it may not offer complete effectiveness against emerging SARS-CoV-2 variants (93, 94). Furthermore, the use of oral antiviral therapies like Paxlovid may carry a risk of COVID-19 rebound syndrome (95) and increased vulnerability to resistance mutations in molnupiravir (96–98).

### 5.2 Use of currently available medications

Given the urgent need for readily available treatments, repurposing already approved drugs can be an effective approach. Furosemide, a loop diuretic, is commonly used to treat edema after congestive heart failure, liver failure, or renal failure (99). It has also been found to have a broad inhibitory effect on the release of pro-inflammatory cytokines such as IL-6, IL-8, and TNF-α (100). Additionally, it has shown potential in reducing the M1 phenotype of microglial cells while upregulating the M2 phenotype (101), suggesting its possible use in treating neuroinflammation in AD. Numerous studies, including those focusing on loop diuretics, have indicated a lower risk of dementia in AD (102–104). These findings provide a promising opportunity for further research and development of molecules targeting neuroinflammation.

### 5.3 Possible future therapies

Although there is currently no standardized treatment for COVID-19, various drugs have shown promise in improving the long-term clinical symptoms of COVID-19 and neurodegenerative diseases. On one hand, the toxic effect on nerve cells can be mitigated by either blocking the long-term influx of Ca^2+^ ions or maintaining internal Ca^2+^ homeostasis. N-methyl-D-aspartate antagonists like amantadine and memantine can achieve this by blocking the extrasynaptic N-methyl-D-aspartate receptors, which weakens the long-term influx of Ca^2+^ ions that contribute to neuronal excitotoxicity. Amantadine, an antiviral drug, has been shown to modestly improve impaired motor behavior in patients with PD and may also reduce fatigue or chronic fatigue. On the other hand, memantine may help improve cognitive deficits. The failure to address these issues can lead to neuronal death and associated functional deficits. Amino adamantane has the potential to be a future therapy for enhancing short- and long-term outcomes of COVID-19 (105). Additionally, lithium can inhibit the upstream pathology of Ca^2+^ dysregulation in both AD and COVID-19 by restoring intracellular Ca^2+^ homeostasis, and it could potentially be repurposed to treat AD patients suffering from COVID-19. Currently, a double-blind, randomized, placebo-controlled trial is recruiting participants to evaluate the efficacy of oral lithium (35–40 mg/day), which has shown greater symptomatic benefit compared to dosages of 10–15 mg/day previously assessed among 50 patients with long COVID (106). However, the effectiveness and risk-benefit analysis of lithium for patients experiencing neurological symptoms due to long COVID have not been established (107).

An alternative therapeutic approach for addressing COVID-19-mediated neurodegeneration is to target neuroinflammatory mechanisms (108–110). The phosphorylation status of ERK1/2 has a positive correlation with viral load. Therefore, inhibiting ERK1/2 can hinder viral replication and infection, by interfering with the binding of SARS-CoV-2 S protein and ACE2 or by inhibiting excessive inflammatory cytokine storm and resistance. Molecules that block ERK1/2 phosphorylation have the potential to prevent viral entry and infection. Naltrexone possesses anti-inflammatory and ERK1/2 inhibitory properties, which can inhibit the binding of receptor binding domain to the host receptor ACE2 (111). Additionally, low-dose naltrexone (LDN) has also demonstrated its ability to inhibit ERK1/2. As a host-targeted broad-spectrum antiviral therapy, naltrexone shows promise in combating COVID-19 infection. Further *in vitro* and *in vivo* studies are necessary to determine the efficacy and understand the molecular basis of these compounds’ anti-coronavirus activity or inhibitory potential (112).

Immunotherapy is an important area of study. It is well established that the vitamin D signaling pathway plays a role in regulating both innate and adaptive immunity, as well as controlling inflammatory responses within normal limits. Vitamin D has pleiotropic immunomodulatory effects and can influence various immune cells at different stages of the immune response. This is achieved through its interaction with the vitamin D receptor, which is expressed in immune cells including polymorphonuclears, macrophages, dendritic cells, and B and T lymphocytes (111). Based on its immunomodulatory properties, vitamin D can be considered as a potential adjuvant therapy for COVID-19. For instance, in a study by the authors of (113), the effects of oral vitamin D supplementation were investigated in patients with mild to moderate COVID-19 and low vitamin D levels. The study found that a dosage of 5,000 IU of vitamin D reduced the recovery time associated with symptoms such as coughing and loss of taste and smell. These findings suggest the potential use of vitamin D in the treatment of COVID-19.

### 5.4 The value of traditional Chinese medicine

Traditional Chinese medicine has been recognized for its significant role in the treatment of COVID-19 sequelae. Studies have shown that flavonoids and chalcones can combat SARS-CoV-2 infection, long COVID-19 disease, and neurodegeneration (114). To target multiple aspects of the disease, researchers have developed multifunctional flavonoid derivatives that can bind to various molecular targets associated with neural changes observed in long COVID-19 disease. Additionally, flavonoids have been found to induce the expression of Nrf2, a protein with tissue and cytoprotective properties that can address issues related to long COVID-19 disease, such as inflammation and hemolysis (96). This can be further enhanced when combined with other novel drugs. Ginkgolides and bilobalide (BB), which are bioactive components of Ginkgo biloba extract, have shown neuroprotective effects in AD through mechanisms such as anti-excitotoxicity, anti-inflammatory, and anti-oxidative activities. Furthermore, ginkgolides and BB may also exhibit antiviral properties against COVID-19 by inhibiting the SARS-CoV-2 main protease. However, it is yet to be determined whether long-term administration of pure ginkgolides or BB at potentially therapeutic levels is truly effective or toxic in the treatment of both AD and COVID-19 (115).

The primary step for patients with neurodegenerative diseases is to actively treat the underlying disease. For individuals experiencing long-term COVID-19 symptoms, it is crucial to focus on early prevention and monitoring. Current treatment approaches involve utilizing existing drugs and actively addressing the disease. Simultaneously, efforts should be made to control inflammation and develop new drugs, including those that can block neurotropic effects and target inflammation. Moreover, extracts from Chinese herbal medicines have exhibited promising anti-inflammatory and antiviral properties, suggesting their potential as innovative and safe treatments for COVID-19.

## 6 Outlook

Long COVID is believed to be an idiopathic disease resulting from chronic inflammation and an exaggerated immune response. The current body of evidence indicates that COVID-19 has long-lasting effects on the nervous system. FDG-PET imaging can provide a more accurate assessment of the severity of patients with long COVID. Treatments for long COVID are still being actively investigated.

The direct relationship between SARS-CoV-2 and neurodegenerative diseases lacks direct pathophysiological evidence. However, further in-depth discussions and subsequent trials are necessary to identify potential targets and develop effective treatments. One promising approach to assess brain involvement in long-term COVID patients is brain FDG-PET, which could aid in the development of different prognostic and management strategies. Additionally, brain FDG-PET can help differentiate clinical symptoms associated with neurodegenerative diseases. To investigate the association between neurological long-term COVID and neurodegenerative diseases, longitudinal follow-up studies are needed. Although there are currently few reported cases of new neurodegenerative diseases after COVID-19, the increasing number of long COVID patients suggests a potential rise in such cases in the future. International collaboration is crucial to gathering more reliable clinical evidence for prevention and follow-up treatment. Furthermore, special attention should be given to the elderly and immunocompromised patients, as they are more susceptible to the long COVID.

## Author contributions

JZ: Writing – original draft. FX: Data curation, Writing – review & editing. XJ: Project administration, Writing – review & editing. XL: Writing – review & editing.
